# Geographic and Temporal Access to Basic Banking Services Offered through Post Offices in Wales

**DOI:** 10.1007/s12061-021-09386-3

**Published:** 2021-08-10

**Authors:** Andra Sonea, René Westerholt

**Affiliations:** 1 Warwick Institute for the Science of Cities, University of Warwick, Mathematical Sciences Building, Coventry, CV4 7AL UK; 2 School of Spatial Planning, TU Dortmund University, August-Schmidt-Straße 10, 44227 Dortmund, Germany

**Keywords:** Basic banking services, Accessibility, Financial inclusion, Distance isochrones, Time capacity

## Abstract

Access to ‘universal banking services’ through the post office network has been a goal of the UK governments over the last twenty years. Various policies and mechanisms have been put in place in an attempt to maintain national geographical coverage with access points while increasing the financial viability of the network. One such mechanism is represented by the six official criteria for access to post offices, expressed as a percentage of the UK population living within one mile, three miles, and six miles of a post office. The method for calculating compliance with these access criteria is not published. Nor will any granular results be published, but only an annual statement that the criteria are being met. This article examines geographical and temporal access to post offices in order to understand the territorial coverage of the network and the impact this has on the provision of basic banking services. The area under investigation is Wales, for which we are reviewing the Government’s official access criteria. Through the Post Office Ltd website, we are collecting up-to-date information on the locations and opening hours of post offices in Wales. In addition, a detailed population grid is combined with calculated areas of equidistant geographical access, called isochrones, to determine the number of people who have access to the post office network. The isochrones are based on the Welsh road network and are calculated for different travel modes and thresholds using a powerful routing engine. Our results show that the official access criteria are largely unmet in Wales. In addition, and in contrast to previous studies, we show a rural-urban divide not in terms of spatial access, but in the combination of spatial and temporal access. The results are of both practical and theoretical value and will hopefully inform policy makers.

## Introduction

The Post Office Ltd is a legal entity owned by the UK Government. This organisation maintains around 11,500 access points and for years it has been used by the UK Government for the exclusive distribution of welfare payments. For these reasons, Post Office Ltd was considered the appropriate vehicle by many UK governments for achieving the financial inclusion of those with no or insufficient access to banking services. A first government policy paper published in the year 2000 recommended that Post Office should develop a concept for a ‘Universal Bank’ (Performance and Innovation Unit, 2000). This idea, however, morphed by 2003 into the quite different concept of ‘universal banking services’ (Midgley, 2005). As a result, the defacto mechanism for delivering such universal banking services is through basic banking accounts with banks and building societies, and serviced either through the banks’ own networks or through a partnership with Post Office. Numerous transformation initiatives and policy reviews have tried to find a viable financial model for the Post Office to deliver basic banking services (Performance and Innovation Unit, 2000; Trade and Industry Committee, 2007; Business and Enterprise Committee, 2008; Public Accounts Committee, 2009).

The UK Government classifies the post office services, including basic banking services, as ‘services of general economic interest’. These are services considered to be of particular economic importance to citizens but which cannot be supplied by market forces alone and require public intervention. This classification allowed the UK Government to seek the authorisation of the European Commission for providing *£*2 billion state aid for the transformation and modernisation of the post office network between 2011 and 2018. The authorisation was obtained on 23 March 2011 (European Commission, 2011) and in return, the Post Office was required to maintain a network of at least 11,500 branches and continue to meet the strict government-set access criteria that see, for example, 99% of the population living within three miles of a Post Office outlet. Providing basic banking services was not optional for the post offices and was now mandated (UK Department for Business Innovation & Skills, 2015). Over the years, the obligation of the Post Office to provide basic banking services and the methods for compensation for these services were regularly enforced and specified in ‘Entrustment Letters’ of the government departments in charge of Post Office Ltd (Lamb, 2012; Swinson, 2015; Callard, 2018). The basic banking services provided through the post office network and which are classified as ‘services of general economic interest’ are: cash withdrawals, cash or cheques deposits using a paper slip specific to the client’s bank, and account balance check.

As revealed in a 2019 public enquiry on ‘The Future of the Post Office Network’ (Business, Energy and Industrial Strategy Committee, 2019c), the post office network found itself again under strain. This was due to service digitisation, postal services deregulation, the loss of welfare distribution by the Department for Work and Pensions, and, among other things, the imposed framework for the provision of banking services for fees that do not cover costs (National Federation of Subpostmasters, 2019). As over 98% of all post offices are run by private businesses often in conjunction with a convenience store or a newsagent, keeping such outlets open depends on their owners’ ability to run a profitable business from that particular location. These businesses are under the legal obligation to serve the customers of the retail banks. However, the banking services are time consuming and often risky, and do not cover their costs for providing these services. “[O]ver 2 500 post office locations out of the total of 11,500 might close in the next 12 months” stated the National Federation of Subpostmasters during a 2019 public enquiry. Twelve months from this statement we do not know if such closures took place and the COVID-19 lockdown rules currently mask the situation.

The number of banks and building societies’ branches decreased over the years from 12,675 in 2015 (Rhodes, 2019) to approximately 7,500 in 2019 (Sonea et al., 2019). Combined with these closures, the likely unsystematic closures of post offices may lead to geographic areas where both post offices and bank branches close simultaneously, potentially affecting access to basic universal banking services for parts of the UK population. The Access to Banking Standard (British Banking Association, 2017) requires banks, in the event of branch closures, to inform affected communities about alternatives and access to them. Such communication customarily indicates that the 11,500 post offices are available for everyday banking. On this balance between the banks and post offices, the Minister in charge of postal affairs, Kelly Tolhurst, MP, stated during the 2019 public enquiry that “Banks have retreated, and sub-masters are picking up the slack” (Business, Energy and Industrial Strategy Committee, 2019b). It is thus of particular relevance for the provision of universal banking services in the UK that the official criteria for access to post offices stand.

The UK Government publishes annually a report on the state of the post office network including the access criteria (Booth & Foley, 2019). These criteria have not changed since their introduction in 2007 (Trade and Industry Committee, 2007):

1. 99% of the UK population shall live within three miles of their nearest post office;
2. 90% of the UK population shall live within one mile of their nearest post office;
3. 99% of the UK population in deprived urban areas shall live within one mile of their nearest post office;
4. 95% of the urban UK population shall live within one mile of their nearest post office;
5. 95% of the rural UK population shall live within three miles of their nearest post office;
6. 95% of the population of every postcode district shall live within six miles of their nearest post office.

These criteria provide a framework for the geographic distribution of post offices offering basic banking services for the customers of those retail banks that are covered by the UK Banking Regulatory Framework (Gola & Roselli, 2009). Both, the access criteria listed above and the individual basic banking services provide the framework into which this article is embedded.

In this article, which focuses on Wales, we investigate the impact of two aspects of the policies and resulting transformations on the goal of achieving a basic financial service provision by Post Office branches: the geographic accessibility to post offices as points of service and their temporal capacities to provide basic banking services. The yearly government report on the access criteria lacks granularity below the six statements given above and the methodology for their calculation is absent. This article shows that the official criteria obfuscate the regional problems of access, as is shown for the case of Wales. Instead of Euclidean distances that are most likely assumed by the government criteria (Citizens Advice, 2017), we propose the use of isochrones similar to catchment areas, which are based on different kinds of real-world street network distances and travel modes. We argue that this provides a more relevant measurement of distance and time-based accessibility with respect to people’s everyday mobility, especially for regions with many natural barriers (e.g., mountains, large lakes, rivers) like Wales, which is covered in proportion of 20% by natural parks that often are barriers in terms of accessibility. In addition to using distance and temporal thresholds for the isochrones, we further construct a simple yet effective capacity measure to reflect the opening hours of post office branches. These capacity measures are further investigated by means of two kinds of spatial hotspot statistics. These shed further light on the performance of the Welsh post office network with regards to providing basic banking services. Our results obtained suggest that the use of more sophisticated spatial analysis can bring answers to questions like the ones raised in the recent public enquiry on the future of the post office network (Business, Energy and Industrial Strategy Committee, 2019a) and may lead to a generally better provision of access to universal banking services.

## Literature Review

The accessibility of critical services and resources as well as everyday facilities has been researched in a number of contexts. These include access to sports facilities (Billaudeau et al., 2011; Higgs et al., 2015; Shrestha et al., 2019) supporting social and societal participation, access to healthy food (Dai & Wang, 2011; Aggarwal et al., 2014; Chen, 2019; Hu et al., 2020) preventing the formation of food deserts and supporting the reduction of obesity, diabetes and other diet-related diseases, and further areas like primary health services (Bauer et al., 2018; Ouko et al., 2019; Taylor & Pettit, 2020), fire stations (Kiran et al., 2020; Shahparvari et al., 2020), residential care facilities (Cheng et al., 2012; Tao et al., 2014; Wang et al., 2020), among others. Accessibility in most of these areas is characterised by a certain degree of homogeneity of what the associated sites and facilities provide. In contrast, measuring access to basic banking services, as examined in this article, is more complex. Access points need to be differentiated not only in terms of temporal capacity (opening hours) and capability (services offered), but also in terms of the wide range of options for satisfying different types of financial needs. Furthermore, financial technologies and consumer behaviours are changing, which cannot be said in the same way for many other facilities of daily use and life, such as sports facilities or places providing health services.

The evaluation of spatial access to post offices has been of recurrent interest in the academic literature. Shortly after their publication, Comber et al. (2009) questioned the government access criteria of post offices, which are also discussed in this present article. The work of Comber et al. (2009) was not only a response to the introduction of these access criteria, but also to the almost simultaneous announcement of the UK Government to close 2,500 post offices. Genetic grouping algorithms (Falkenauer, 1998) were used to show that alternative closure plans would have led to a lower impact of these closures on people with a higher need for accessible post offices, such as people with long-term illnesses or unpaid carers with extensive care responsibilities. In a follow-up study, Comber et al. (2012) examined individual perceptions of accessibility to public services. Using surveys and Geographically Weighted Regression (Fotheringham et al., 2003) the authors concluded that geographical distance is a strong predictor of dissatisfaction with access to post offices. They also found that the effect of geographical distance is strongly dependent on socioeconomic status. The results reported by Comber et al. (2009, 2012) refer to Leicester and Leicestershire. Using the same research area and also focusing on branch closures, Ibrahim and Lawal (2015) conclude that most government criteria for access to post offices are not met in either the urban areas of the City of Leicester nor in the surrounding rural county.

Further studies on access to post offices have been carried out to investigate access on a larger scale. Some of these studies explicitly refer to our area of study: Wales. In one study focusing on potential differences between urban and rural areas, Langford and Higgs (2010) reported no systematic discrimination against rural dwellers in terms of access. Rather, they found that the perception of who is most affected by closures depends largely on the geographical distance measure used and that the perception of an urban-rural divide can be caused by the use of Euclidean distance measures. However, what this particular study could confirm is that the government criteria for access to post offices in Wales are generally not met. The results therefore support the local findings set out in the previous paragraph. Also focusing on Wales, Higgs and Langford (2013) examined the impact of the closure of 2,500 post offices on access to post offices for the older population. This study also uses an urban-rural perspective and, like Langford and Higgs (2010), cannot confirm systematic differences between the two types of statistical output areas in terms of demography. Also, in a very recent study, Langford et al. (2020) have investigated access not to post offices but to retail bank branches in Wales. Given the recency of this study, and to provide a timely and full picture on the situation in Wales, we will compare our results obtained for the post office network to their results in the conclusions section.

Both studies, Langford and Higgs (2010) and Higgs and Langford (2013), show similarities with the research presented in this article. A key difference between the present study and the earlier studies mentioned is that we, apart from our more recent database, do not exclusively rely on statistical output areas. Instead, in addition to using the 2011 Output Areas provided by the Office for National Statistics, our methodology includes the use of a granular population grid, which allows detailed population estimates also in very rural areas. However, the latter is at the expense of not being able to differentiate socioeconomically and demographically. Another difference to previous works is that we calculate network distances on the basis of OpenStreetMap data, which for the present case of Wales, and similar to most parts of Western Europe, shows a completeness of more than 90% (Barrington-Leigh & Millard-Ball, 2017). Combined with a powerful routing engine, this allows us to calculate routes for different modes of transport, including pedestrian routes using footpaths and tracks, and to take into account one-way restrictions, intersection details, and other traffic-related rules, provided these have been mapped by the OpenStreetMap community.

## Data

### Post Office Locations

One of our main data sets for the present analysis is a database of post office locations. The locations, types, and opening hours of post offices are constantly changing. However, there is no way to monitor this information in an automated fashion, as is the case with the network of bank branches of the major banks in the UK, for which we have open banking APIs (Open Banking Implementation Entity, 2017). Thus, there are a number of different but inconsistent ways of collecting post office locations:

1. The official locations offered by Post Office Ltd on their website (no longer available online). This data set dates March 2018 and contains postcode-level detail only, but no other information about the locations offered (Post Office, 2018). According to this data set, there are 906 post offices in Wales.
2. A data set obtained through a Freedom of Information Request dating February 2020 (Post Office, 2020). This data set (which is no longer available online) lists 953 post offices across Wales, but information about the types of the offices, their services offered, and their opening hours are missing.
3. The latest Post Office Numbers Report for 2019 (Foley, 2020) states that there are 931 locations for Wales. Yet, neither the report nor accompanying data disclose any actual locations.
4. A data set web-scraped by us, for the whole UK, from the Post Office website^1^ in July 2019. Out of the whole data set we have preserved for the purpose of our analysis a number of 1,202 post offices out of which 846 are in Wales and 356 within a 20 km buffer around Wales’ borders. This was necessary in order to calculate correctly the isochrones for the areas in Wales which border England, since people resident there can also use those English branches.

**Fig. 1:**
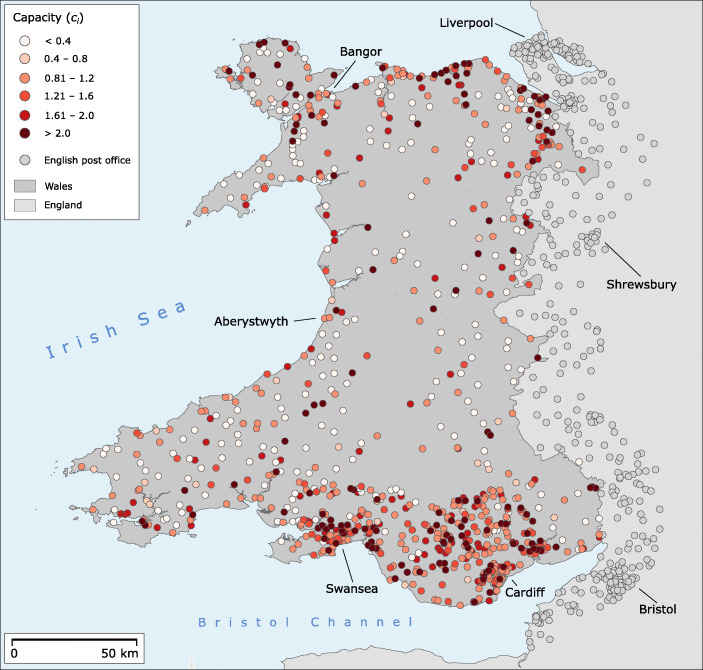
Overview of all Post Office locations in Wales and nearby parts of England. The capacities *c*_*i*_ introduced in “Branch Capacity” are illustrated for the Welsh post offices

This variability between data sets shows that the post office network is dynamic and changes over time. While we can identify differences between some of the data sets and observe that some offices close while others open elsewhere, we do not have information about the drivers of these changes. For this paper, we work with the web-scraped data from the Post Office’s website collected in July 2019 because we assume this data set to reflect best the actual experience of somebody looking for a nearby post office for basic banking services. We are thus able to use the latitude and longitude of the post offices and also their opening hours and lists of services offered. The opening hours are used as a measure of capacity of a location, while the list of services is used for assessing the finance related services provided by a post office. The ‘Finance’ services at the time of the extraction were limited to: ATMs open 24 h, Current Account Servicing, and Savings ID Verification. Our web-scraping tool based on Python and BeautifulSoup retrieved the following information: name, telephone, address, postcode, latitude, longitude, opening and closing times for each day of the week, and a list of services including Finance services. While the latitude and longitude are not directly visible on the web page they are included in JSON structures that are used in the pages to build the embedded web maps showing each post offices’ location. The scraped post office locations used in the remainder are visualised in Fig. 1. The annotated code used for web-scraping is provided as online supplementary material. None of the data sets listed above contains the types of post offices at individual location level: Crown, Agency, Outreach. These different types of post offices vary considerably in terms of services offered (see Booth & Foley 2019). For future research, it would be useful to also consider this dimension as the type of the post office is indicative of the size and setting of a location and hence its capability to provide banking services. The type of post office is also likely to have an impact on how people perceive their own access to these services. At present, however, we are not aware of any publicly accessible data set detailing the types of post offices at this level.

### Population Grid

**Fig. 2:**
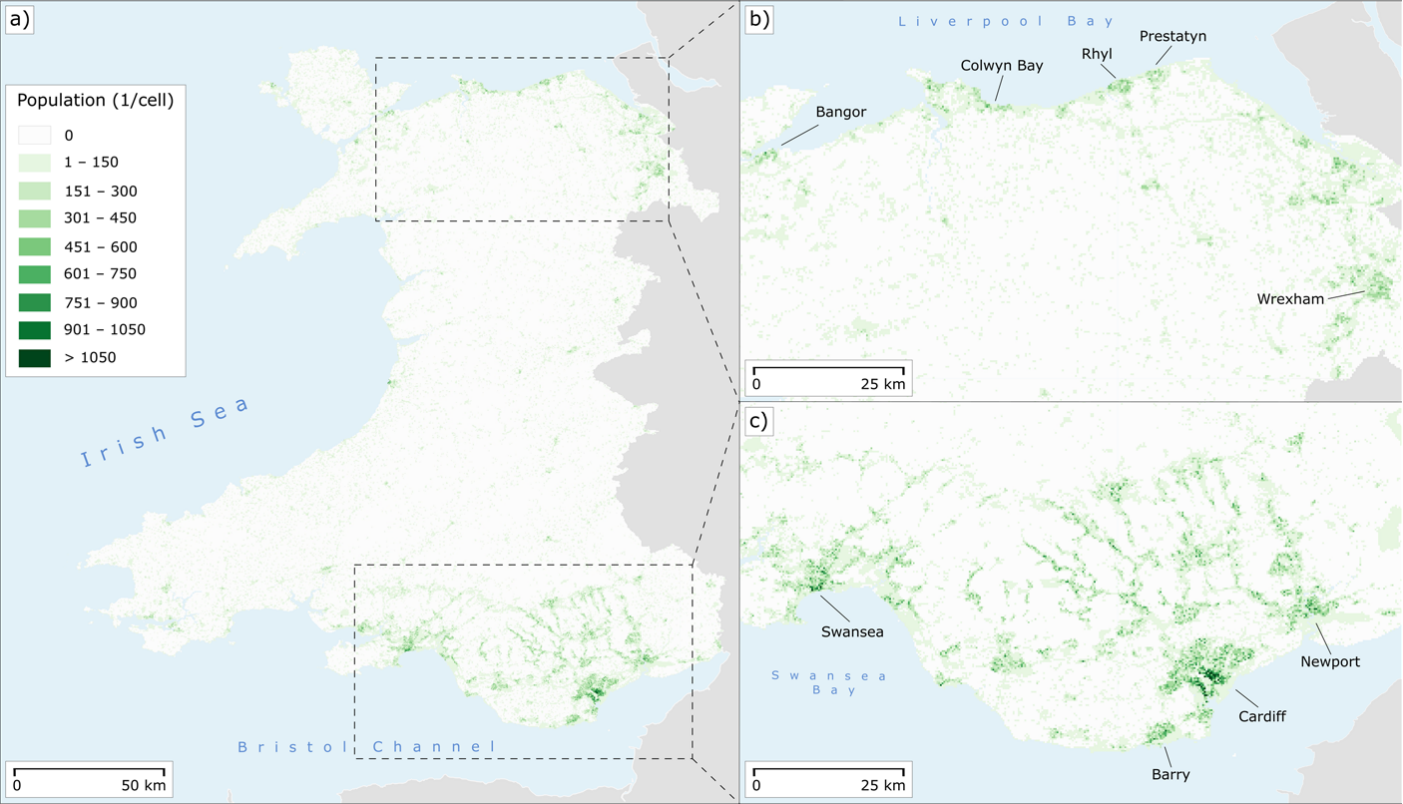
Population distribution in Wales on a 250-m grid taken from the European Commission’s Global Human Settlement Initiative. **a** Overview of the entire country. **b** Close-up map of the northern populated coastal areas. **c** Close-up map of the South Wales conurbation

An estimated population grid is used to determine the number of people who have access to post offices. The data set used is taken from the European Commission’s Global Human Settlement Initiative, which was carried out together with Brazilian, Chinese and South African institutions. One of the layers produced by this initiative is the GHS-POP layer, which provides a global population grid with a resolution of 250 m (Florczyk et al., 2019). The grid is estimated in a process using both satellite imagery and census data. First, built-up areas were extracted from Landsat-8 satellite images. This step is based on the use of symbolic machine learning (Pesaresi et al., 2016), whereby geometrically more accurate but not globally available Sentinel-1 data was used to train the classifier. The result is the so-called GHS-BUILT layer, which provides the proportions of built-up area for each grid cell. These extracted proportions were then combined with local census data sets by disaggregating the available census units using dasymetric mapping according to the proportion of built-up area in each grid cell. The data used for Wales are the 2011 census figures extrapolated to 2015 using growth rates calculated ceteris paribus from previous censuses (Doxsey-Whitfield et al., 2015). The complete procedure is outlined in Freire et al. (2016) and Corbane et al. (2019). We have used the 2019 revision of the GHS-POP layer, which is based on more accurate input data than the original grid. A visual comparison between both versions has shown that the newer version contains less inaccuracies especially in the rural areas of Wales, where sometimes open rock or bare earth are wrongly classified as built-up area in the original grid. The map in Fig. 2 provides an overview of the population grid for the study area in Wales including close-up views of the more urbanised parts of the country in Fig. 2a and b. We argue that this population data set is more accurate than some alternative methods like assuming a homogeneous population structure across entire LSOAs as used, for instance, by Comber et al. (2009). The latter may work well in smaller, relatively homogeneous areas (such as Leicestershire), but is likely to be inaccurate, particularly in the more rural parts of Wales characterised by disparities between settlements confined in valleys and largely uninhabited surrounding areas. However, it is important to bear in mind that the GHS-POP layer is a collection of estimates. The latter are naturally susceptible to estimation errors, for example due to misclassifications of the Landsat imagery or incorrect assignments of census populations to the identified built-up areas. The grid may thus be more accurate in rural areas than many alternatives, but its use is by no means without limitations. For example, a quarry near the town of Rhiwbryfdir was incorrectly classified as built-up area, although misclassifications of this size are rare. In the densely populated parts of Wales, the raster used may even be less accurate than fine-grained census units. For this reason, we have based parts of our analysis not only on the raster described above, but also on Output Areas, the most fine-grained British census unit. In this way, combining utilisation of both population data sources, we hope to obtain a comprehensive picture of both rural and urban areas.

### Output Areas

In addition to the population grid outlined above, Output Areas,^2^ the lowest geographical level of the 2011 UK census, were used in our analysis. The current tessellation is very similar to the units used in the 2001 census, and only about 2.6% of all Output Areas have changed since then. The minimum size of Output Areas is 40 resident households or 100 residents, while the target size is 125 households per area. Wales is covered by a total of 10,036 Output Areas. We have used the mid-2019 population projection^3^ based on an extrapolation of the 2011 census, estimating Wales’ population at 3,152,870. The estimate is used to be temporally consistent with our Post Office data set, which was scraped in the same year. The use of census units such as Output Areas is particularly useful for looking into urban areas, where coverage is quite dense due to the characteristic of census units to cover a comparable number of households. However, as explained in the previous section, this is not always the case in rural areas. Another general problem with census units such as Output Areas is that they are designed according to demographic rather than social or geographical criteria. This may lead to problems in some parts of the country in relation to the Modifiable Areal Unit Problem (see Openshaw 1983; Bluemke et al., 2017). The population numbers were determined from the Output Areas in combination with calculated accessibility isochrones (see Isochrone Calculation). For this purpose, the share of the population of an Output Area is assigned proportionally according to the intersection with the isochrone, whereby for each isochrone, all overlapping Output Areas are summed up accordingly.

## Methodology

Accessibility measures for public services can be classified into four groups (Geurs and Van Wee, 2004): infrastructure, location, person, and utility-based. Each of these groups consist of four main components: transport, land-use, temporal and individual. For the purpose of this article, we consider location and time-based measures, where we calculate a travel time or cost between locations distributed spatially.

### Isochrone Calculation

**Fig. 3:**
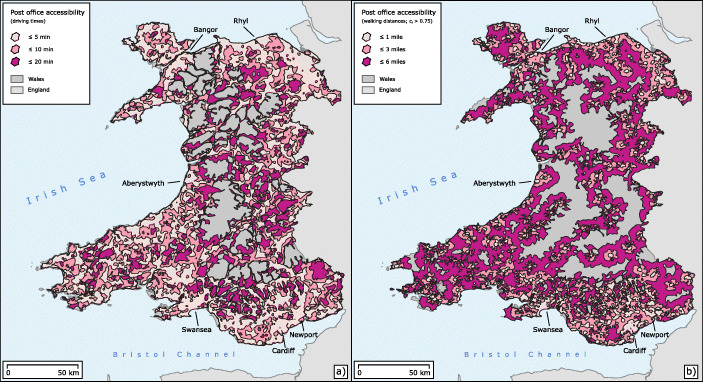
Illustration of the isochrones used for distance modelling. The isochrones shown have been joined to facilitate better visualisation. **a** Car and time-based isochrones representing all post offices. **b** Pedestrian and distance-based isochrones of post offices with *c*_*i*_ > 0.75

An important part of our methodology is the reliable measurement of geographical distances between post offices and their potential customers. It would be technically cumbersome to calculate such geographical routes on a household basis. We therefore use an inverse approach based on isochrones, using post offices as starting points. An isochrone is a territory bounded by a line connecting equal network-derived distances or times from a given location. In this way, we can get a good approximation of the way people make journeys in everyday life, which, in the case of regular trips, is largely determined by the time needed and the choice of mode of transport (O’Sullivan et al., 2000; Dovey et al., 2017). Put simply, people know approximately how long it takes them to get to a particular local point of interest by foot, car, or public transport, and they make their decisions on combining trips based on this local information. Figure 3 illustrates two types of isochrones used in this article, car and walking-based as well as with and without taking account of temporal capacities as introduced below in “Branch Capacity”. Further isochrones are provided as interactive web maps, the links to which can be found in the section on supplementary material.

Isochrones react sensitively to distance and time thresholds. They can therefore vary considerably in size and shape depending on the thresholds used, which affects the measurements of accessibility (Xi et al., 2018). Without having suitable time thresholds at hand for the case of post office access, we calculate the time isochrones for 5, 10, and 20 min for both walking and car mode. This choice is based on walking distances from the public transport literature where thresholds of about 20 min have been reported as an upper bound (see Table 2 in van Soest et al., 2020). We further argue that the values chosen reflect useful time thresholds that many people would be willing to spend on a regular task like banking. We do not use different temporal thresholds for motorised and non-motorised modes, as the choice of mode does not affect the amount of time that would be considered acceptable. In addition, we calculate distance isochrones, which allow us to compare our results with the 1, 3, and 6-mile distance thresholds from the official access criteria of the Post Office. This was done for both walking and driving modes, because these take into account different kinds of paths (e.g., footpaths for non-motorised routing vs motorways for car-based routing). We have used the Openrouteservice of the Institute of Geography at Heidelberg University in Germany for the implementation of our isochrones (Neis & Zipf, 2008; Heidelberg Institute for Geoinformation Technology, 2020). This web service offers a freely available API and allows for the calculation of isochrones on the basis of frequently updated OpenStreetMap data.

### Branch Capacity

Access to the post office services is not only spatial in nature, but also has a time component reflected in the opening hours of branches. Research has shown that the provision of appropriate opening hours influences the actual and perceived accessibility of public services and of services of public interest (Neutens et al., 2012; Cowling et al., 2018). The strength of this influence varies for different types of services. For quotidian services like basic banking, however, the opening hours have been shown to be important factors (Chen & Clark, 2016; Järv et al., 2018). Our study area of Wales further is a predominantly rural part of the United Kingdom with a rather inaccessible topography. This implies an uneven and fragmented population distribution, which, in turn, may affect the profitability of some of the privately run post offices. For these reasons, and because we understand accessibility as “the potential of opportunities for interaction” (Hansen 1959, p. 73), we use a simple but meaningful measure of post office capacity in addition to the isochrones described above. Assuming *t*_*i*_ to be the total opening time in minutes per week, the capacity measure *c*_*i*_ results from the ratio *c*_*i*_ = *t*_*i*_/2760. The division by 2760 reflects a comparison with the usual opening hours for standard bank branches (that is, full-time stationary branches) in minutes per week (see Sonea et al., 2019).

**Fig. 4:**
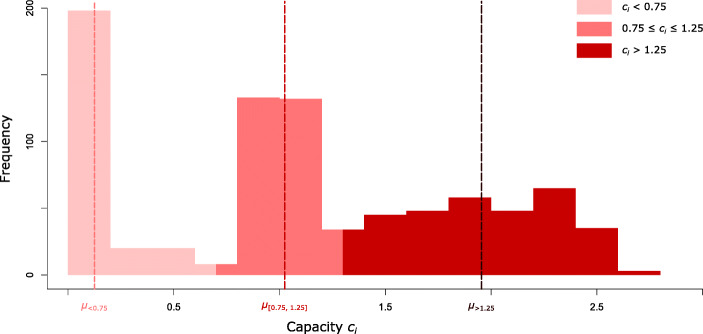
Histogram of the *c*_*i*_ capacity values. The dashed vertical lines indicate the mean values of the three subsets of the capacity values that are examined separately in this article

There are 243 postal offices in our data set with *c*_*i*_ < 0.75 which include 120 post offices open less than 2 h per week. When calculating isochrones, we, on the one hand, do this for all access points and, on the other hand, separately only for those with *c*_*i*_ > 0.75, which most likely are perceived as permanent post office locations offering adequate service times. We also identify post offices with *c*_*i*_ > 1.25 in order to observe opening hours patterns of those offices offering above-average temporal service. The very long opening hours of this last category usually indicate that post office services are offered inside convenience stores and retail outlets, which by no means indicate a wider or better banking services offering beyond temporal concerns. The post offices with a capacity on the interval [0.75,1.25] are considered comparable to a stationary retail bank branch with normal opening hours. To the best of our knowledge, there is no easily obtainable data set reflecting the exact opening hours specifically for banking services inside a post office. We therefore consider our capacity figures to be a good approximation to those exact times, but with the limitations outlined. The mapped capacity values for Wales are found in Fig. 1. In addition, Fig. 4 provides a histogram of the scores including the separate mean values for the three subsets of *c*_*i*_ values as outlined above. It should be mentioned that, beyond our focus on capacity, it would also be interesting to look into banking capability including the number of service counters available, or the range of services offered. Unfortunately, we do not have this kind of information available but future studies may add this important dimension.

### Exploratory Hotspot Statistics

We apply two exploratory hotspot statistics to the capacity measurements from “Branch Capacity”. Hotspot statistics reveal areas where extremely high (‘hotspot’) or low (‘coldspot’) attribute values are concentrated geographically. One measure we use is the widely applied Getis-Ord $G_{i}^{*}$ (Ord & Getis, 1995) statistic, which is given as

1 $$ G_{i}^{*} = \frac{\displaystyle\sum\limits_{j=1}^{n}{w_{ij}x_{j}} - \bar{x}\sum\limits_{j=1}^{n}{w_{ij}}}{ \sqrt{ \displaystyle\frac{s^{2}}{n-1} \left( n \sum\limits_{j=1}^{n}{w_{ij}^{2}} - \left( \sum\limits_{j=1}^{n}{w_{ij}} \right)^{2} \right)} }, $$

where *w*_*ij*_ are spatial weights between *n* sites *i* and *j*, for which attribute values *x*_*i*_ are available. The constants $\bar {x}$ and *s* are the usual mean and standard deviation of the *x*_*i*_ values. The measure $G_{i}^{*}$ is used here as an indicator for the spatial concentration of the capacity measure *c*_*i*_ across the observation area. This hotspot measure calculates the local spatially weighted sum of the attribute under consideration and standardises it with the global mean and the standard deviation of the statistic, both of which are also estimated taking the spatial structure into account. The measure is approximately standard normal. Therefore, values of $G_{i}^{*} \geq 1.96$ or $G_{i}^{*} \leq -1.96$ respectively indicate a significant local accumulation of either high or low *c*_*i*_ values at a 95% level of confidence, not only at the point locations where the local statistics are recorded but also in the surrounding neighborhoods as defined by the spatial weights. We parametrise the latter with 10-nearest-neighbour weights that well reflect the varying geometric scales between the rural and more urbanised parts of the map. The resulting statistical maps provide an overview of the overall capacity distribution in Wales.

We are also interested in characterising the differences within the urbanised North and South Welsh agglomerations. The statistic $G_{i}^{*}$ cannot afford this, as it uses global reference values such as the mean and standard deviation of the attribute values. These are partly based on observations from rural areas, making it difficult to discern more subtle differences within urban areas. We therefore use *GS*_*i*_ (Westerholt et al., 2015), a variant of $G_{i}^{*}$ taking these differences into account in the estimations of all reference values. The measure is given as

2 $$ \begin{array}{@{}rcl@{}} GS_{i} = \frac{\displaystyle\sum\limits_{j; k < j}{w_{ij}w_{ik}\phi_{jk}\left( x_{j} + x_{k}\right)} - \frac{W_{i}}{{\phi}} \sum\limits_{j; k < j}{\phi_{jk}\left( x_{j} + x_{k}\right)}}{\sqrt{\displaystyle\frac{W_{i}}{{\phi}}\sum\limits_{j; k < j}{\phi_{jk}\left( x_{j} + x_{k}\right)^{2}} + \frac{W_{i}\left( W_{i}-1\right)}{{\phi}\left( {\phi}-1\right)}\left( \!{\varGamma}^{2} -\!\! \sum\limits_{j; k < j}{\phi_{jk}^{2}\left( x_{j} + x_{k}\right)^{2}}\right) - \left( \frac{W_{i}}{{\phi}}\sum\limits_{j; k < j}{\phi_{jk}\left( x_{j} + x_{k}\right)}\right)^{2}}}, \end{array} $$

where the additional binary weights *ϕ*_*jk*_ indicate whether two observations *j* and *k* are linked on a given geometrical scale. The double sums iterate over the lower triangular parts of the matrices involved, with the index *j* running from 1 to *n* and the index *k* from 1 to *j* − 1. The row sums *W*_*i*_ of all weights combined, the overall number of scale-fitting relationships *ϕ*, and the total of the pair-wise sums of attribute values *Γ* of all interactions at the analysis scale are given by

$$ W_{i} = \sum\limits_{j; k < j}{w_{ij}w_{ik}\phi_{jk}}, \qquad {\phi} = \sum\limits_{j; k < j}{\phi_{jk}}, \qquad \text{and} \qquad {\varGamma} = \sum\limits_{j; k < j}{\phi_{jk}\left( x_{j} + x_{k}\right)}. $$

The measure *GS*_*i*_ is also standard normal and has a similar interpretation to $G_{i}^{*}$. The spatial weights used in this measure are binary distance-based weights with a distance threshold set to 2,500 m. The results obtained allow differentiated insights into the local spatial structure of capacity values within urban areas. This makes it possible to assess the influence of temporal capacity on the accessibility of basic banking services beyond the mere global distribution throughout Wales.

## Results and Discussion

### Distance Isochrones and the Official Access Criteria

The official Post Office access criteria are not generally met in Wales. The government estimates that 99% of the population is covered at a distance threshold of 3 miles. As can be seen from the results set out in Table 1, our figures for the 3-miles limit are 92.80% (car) and 94.47% (walking), representing a deficit of about 6 percentage points compared to the official criteria. For the 1 mile threshold our results show that only 72.43% of the Welsh population are able to reach a post office within 1-mile driving distance of their homes. This represents a deficit of more than 17 percentage points compared with what the government assumes for this distance threshold. For pedestrian travel mode the isochrones for the 1-mile threshold cover 77.37% of the population. This looks better than the car-based figure since those isochrones take into account footpaths, lanes, and other routes that are not accessible by car but can be used for walking. However, this figure is still far from the government criterion of 90%, which means that the accessibility of post offices in the immediate vicinity of residential areas in Wales is not sufficiently developed. The 6-mile threshold is an outlier in our comparison. The corresponding isochrones cover 98.38% (car) and respectively 99.23% (walking) of the population, which seems to be above the 95% official criterion for this threshold. The 6-miles figures, however, are not directly comparable because the government uses Euclidean distances to centroids of Postcode Districts in this case.

**Table 1:** Shares of the Welsh population living within certain thresholds from a post office assessed on the basis of the population grid

Mode	Threshold	Official criteria	Isochrones		
			All	*c*_*i*_ ≤ 0.75 only	*c*_*i*_ > 0.75
Car	6 miles	95%[a]	98.38%	0.77%	97.61%
	3 miles	99%	92.80%	2.98%	89.82%
	1 mile	90%	72.43%	3.73%	68.70%
	20 min	N/A	98.82%	0.10%	98.72%
	10 min	N/A	97.54%	1.26%	96.28%
	5 min	N/A	92.01%	3.49%	85.52%
Foot	6 miles	95%[a]	99.23%	0.73%	98.50%
	3 miles	99%	94.47%	3.15%	91.32%
	1 mile	90%	77.37%	3.70%	73.67%
	20 min	N/A	79.04%	3.74%	75.30%
	10 min	N/A	49.15%	3.14%	46.01%
	5 min	N/A	19.28%	1.75%	17.53%

Note that the temporal assessment is not available under the government criteria. Further, the government criteria do not distinguish between different travel modes

^a^ Uses Postcode Districts and is thus not directly comparable with the other measures

The official access criteria and the calculations outlined above consider all post offices to be equivalent. From the population’s point of view, however, there are big differences between access points that are open only a few hours a week and those that offer an almost permanent presence. Therefore, in our analysis we distinguish post offices according to the capacity values we calculated. The figures look favourable for larger distance thresholds. Regardless of the mode of travel, more than 97% of the population have access to post offices with *c*_*i*_ > 0.75, which offer average or better time service, when considering the 6-mile figures. These figures are decreasing, but remain relatively high for the 3-mile distance threshold. However, only 73.67% of the Welsh population can reach a post office with *c*_*i*_ > 0.75 within a 1-mile distance on foot from their home. At the same distance, the percentage is even lower when looking at the figures for car journeys, which is an effect of the different types of paths included in the routing procedures (that is, the pedestrian routing also taking into account footpaths, tracks, etc.). Table 1 also gives results for the exclusive access to post offices with *c*_*i*_ ≤ 0.75 that can be classified as undersupplying. For both mobility modes, the results for the 1 and 3 mile ranges, which are particularly relevant to everyday life, show that around 3 to 4% of the Welsh population are consistently under-served in terms of basic banking services offered through post offices. This is equivalent to around 125,000 people, equivalent to half the size of Swansea, the second largest city in Wales. It is also worth noting that the capacities of the vast majority of post offices with capacity values below 0.75 are close to 0, as can be seen from the histogram in Fig. 4. The mean value of the capacities below 0.75 is 0.12 (corresponding to 5.52 h/week) and the median is only 0.05 (2.3 h/week). These strong imbalances between the capacities of post offices will be further examined spatially in “Geographical Exploration of the Temporal Capacities”. What the results presented in this sub-section show is that the pedestrian accessibility of post offices in Wales, which are open for a sufficiently long time, is a significant problem. This is further supported by the isochrones in Fig. 3b, which visualise pedestrian accessibility of those post offices with acceptable opening hours.

The results of the calculations based on Output Areas are given in Table 2 and they indicate an even lower coverage for both 1 mile and 3 miles thresholds. Only 66.29% of the population in Wales live within 1 mile by car from a post office and 71.95% can reach a post office within the same distance by walking. The percentages are even lower if we consider only the post offices open more than 23 h per week or with a capacity *c*_*i*_ > 0.75 respectively: 63.55% (car) and 69.15% (walking). We note significant differences between the percentages of population calculated using the two population layers: the 250-m grid and the ONS Output Areas. While the two layers have been obtained through different methods themselves, for us the main point of this calculation is to estimate if the access criteria are respected or if indeed these criteria are fit for the purpose of estimating access in an area like Wales. Both calculations show that the access criteria are not met for Wales especially for 1 and 3 miles thresholds. A very likely explanation for the differences between the two population estimates may be the more crude redistribution of the population using the Output Area-based method.

**Table 2:** Shares of the Welsh population living within certain thresholds from a post office assessed on the basis of Output Areas

Mode	Threshold	Official criteria	Isochrones		
			All	*c*_*i*_ ≤ 0.75 only	*c*_*i*_ > 0.75
Car	6 miles	95%[a]	97.69%	0.99%	96.70%
	3 miles	99%	88.76%	2.61%	86.15%
	1 mile	90%	66.29%	2.74%	63.55%
	20 min	N/A	98.54%	0.19%	98.35%
	10 min	N/A	95.83%	1.55%	94.28%
	5 min	N/A	87.72%	3.12%	84.60%
Foot	6 miles	95%[a]	98.87%	0.90%	97.97%
	3 miles	99%	91.67%	0.35%	91.32%
	1 mile	90%	71.95%	2.80%	69.15%
	20 min	N/A	72.18%	2.66%	69.52%
	10 min	N/A	43.43%	2.00%	41.43%
	5 min	N/A	16.28%	0.96%	15.32%

Note that the temporal assessment is not available under the government criteria. Further, the government criteria do not distinguish between different travel modes

^a^ Uses Postcode Districts and is thus not directly comparable with the other measures

### Time-Based Access Criteria

As people customarily think of their journeys in terms of time we look at how accessible the post offices in Wales are within 5, 10, and 20-min journeys by car and walking. The differences between walking and driving accessibility are very marked for time-based measures as can be seen from Table 1. The differences in percentage points between those who have access within 5-min driving time and those with a post office within 20-min driving time are only about 6 percentage points. However, the situation is quite different for pedestrian access. A proportion of 79.04% of the Welsh population have access to the nearest post office within 20-min walking time. Yet, this figure falls significantly to a small percentage of only 19.28% if the threshold is lowered to 5 min. This difference shows that access to a post office in Wales depends greatly either on the availability of a car or on living in the few urban parts of the country. Another important finding is that car-based figures that are based on time thresholds are much closer to the government criteria, although the latter are based on distance thresholds and hence not comparable one by one. One possible interpretation could be that the government criteria may be more geared to the situation in England, where the population is more evenly distributed and less spatially concentrated than in Wales. In any case, we can conclude that pedestrian access to post offices in Wales does not have a high population coverage, in particular with respect to the lower thresholds that are more relevant to everyday life. Realistically, most people in Wales would need to use a car to go to a post office and hence also for doing basic banking.

The proportion of the population living within 5, 10, and 20 min of post offices with good time availability decreases with the thresholds for both driving and especially walking. The results presented in Table 1 show that 75.30% of the Welsh population can walk to a post office with at least average time availability within 20 min. This figure falls dramatically to only 17.53% if the threshold is reduced to 5 min. At the same time, the proportion of those who have access only to post offices with very low capacity levels is similar to the distance-based results. However, here the values are relatively high even for longer walking times. In fact, the proportion of the population having access to poorly served post offices actually increases with the walking time, which is an effect of the fact that many people have no access at all (i.e., also not to post offices with low capacity values) when walking for 5 and 10 min. Nevertheless, these results confirm the results reported in both the previous paragraph and the previous subsection. They show how poorly walkable the post office infrastructure is in Wales and how much the people of Wales need to rely on individual motorised vehicles to reach, in particular, those post offices whose service times are long enough to realistically integrate basic banking services into everyday life.

Using the Output Area-based population estimate, we calculated the population included in the time isochrones for car and walking mode of transport for the 5, 10, and 20 min thresholds. The results presented in Table 2 again show lower coverage than with our calculations using the 250-m population grid. Only 69.52% of the Welsh population lives within a 20 min walk of a post office open more than 23 h per week. The coverage is, however, quite high for all thresholds for those who have a car. This highlights how differently people resident in the same area can experience access to the same locations.

### Geographical Exploration of the Temporal Capacities

**Fig. 5:**
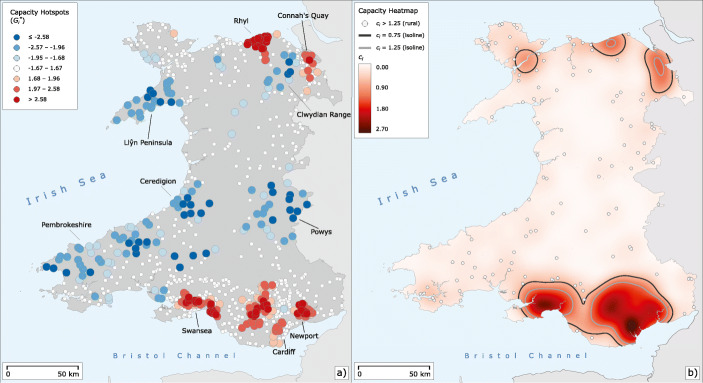
Capacity hotspots in Wales. **a** Getis-Ord $G_{i}^{*}$ z-scores. **b** Heatmap of *c*_*i*_ capacity values with isolines that define spatial areas within which *c*_*i*_ values are often statistically close to 1. The points visualise rural post offices with *c*_*i*_ > 1.25

The temporal capacity indicator is not evenly distributed across Wales. Figure 5a shows a hotspot map based on $G_{i}^{*}$ estimated for *c*_*i*_. There is a striking difference between the more urbanised areas in the north and south and the more rural parts of central Wales. This contrasts with the results for the isochrones, where the coverage of urban and rural areas does not contrast so starkly, though it also exists. Additional support for the pronounced urban-rural gradient is found in the isolines in Fig. 5b. These isolines delineate areas where post offices tend to have service hours similar to those of retail bank branches. As these areas are rather small and located in the suburban peripheries of larger municipalities, the change from better temporal accessibility in urban areas to less convenient capacities in rural areas is rather abrupt. The areas with the strongest hotspots of temporal accessibility are Rhyl, Wrexham and Connah’s Quay in the North, and the urban areas of Swansea and Cardiff including Newport in the South. Surprisingly, the areas with the strongest spatial concentrations of high *c*_*i*_ measures in the South are not the urban centres of the two largest Welsh cities of Cardiff and Swansea, but the surrounding suburban areas in the larger urban agglomerations of these cities. This is most evident in Cardiff, where a large strip immediately north of the city stands out on the map. These effects are not so marked in the North, where municipalities are generally smaller. Interesting exceptions are the city of Bangor and the seaside resort of Colwyn Bay. These are not shown as coldspots, but still deviate from the general urban pattern.

The post offices are generally less accessible in the rural areas of Wales. Much of the countryside is not part of either a hot or coldspot, but is not statistically significant in terms of $G_{i}^{*}$. However, the strong and large coldspots are all located in the rural central and coastal areas of Wales. Based on *c*_*i*_, the least temporally accessible areas are the Llŷn peninsula, the Clwydian Range, some of the south-central areas of Powys, and a coastal strip stretching from Ceredigion to Pembrokeshire. Several interesting patterns can be seen among the coldspots. Looking at the largest cluster of coldspots in Ceredigion and Pembrokeshire, there is a tendency towards a higher spatial concentration of low capacity values in the hinterland of the coastal areas. This pattern does not seem to be caused by a small-scale rural/urban discrepancy, as some of the larger municipalities in the region are located in the coastal hinterland and are part of some of the strongest coldspots on the map (for instance, Newcastle Emlyn). A likely explanation for this is that the coastal areas are more developed for tourism and may therefore offer a slightly better postal infrastructure. This is confirmed by the outlier post offices that can be seen in Fig. 5b. Some of these isolated post offices offering high temporal capacity are located on the coast, while a wider strip in the adjacent inland area does not contain many such post offices. The pattern for Powys is likely to be related to the ring of small towns surrounding a predominantly natural area near the English border where the strongest coldspots in Powys are located. The same interpretation can be made for the Clwydian Range, which is largely uninhabited despite some larger nearby municipalities like Wrexham. Likewise, Llŷn is also sparsely populated, which explains the pattern of low capacity values that are clustered on the peninsula. Another notable observation is that most of the very strong coldspots are associated with *c*_*i*_ values below 0.4 and thus likely with the appearance of mobile post offices. While several of the coldspots are located in sparsely populated areas, follow-up research should qualitatively investigate why some other similarly sparsely populated areas are not identified as coldspots.

**Fig. 6:**
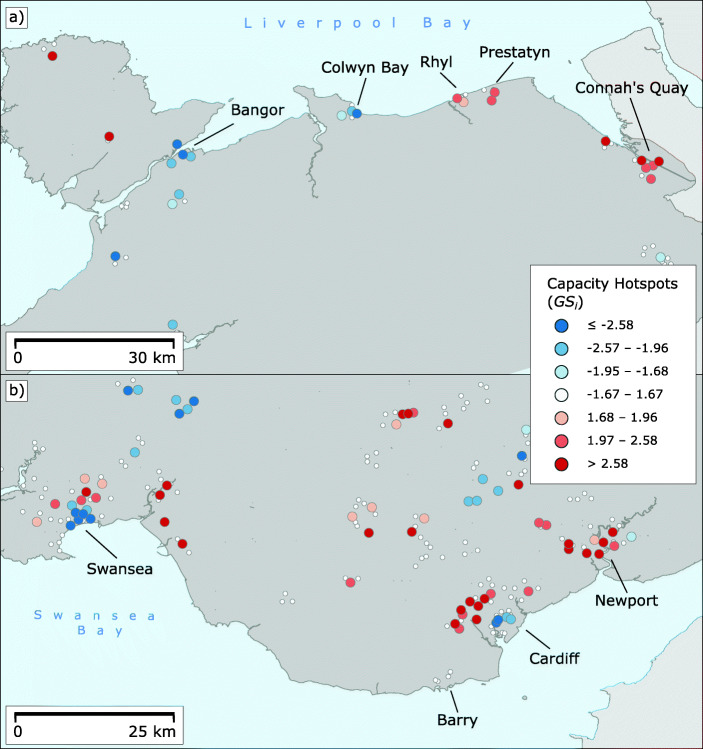
Capacity hotspots in urbanised areas of Wales. **a***GS*_*i*_ z-scores for the North Wales conurbation. **b** *GS*_*i*_ z-scores for the South Wales conurbation

The analysis of the spatial clustering of *c*_*i*_ with a focus on urbanised areas only discloses additional structures in the measurement of temporal capacity. Figure 6 shows maps of *GS*_*i*_ z-scores of *c*_*i*_ for both the northern and southern Welsh agglomerations. The hot and coldspots shown are to be interpreted in relation to other urban areas only, as the rural data are not included in this analysis. The northern part shown in Fig. 6a shows an interesting east-west divide. The cities located in the western counties of Gwynedd and Conwy have high concentrations with low capacity values compared to other urban areas. This includes the city of Bangor, which explains why this municipality does not appear as a hotspot in the $G_{i}^{*}$ analysis like other cities. A closer look at Bangor, but also Colwyn Bay, reveals many independent post offices, often in retail areas. Furthermore, there are more mobile post offices than in the eastern part of the North, which has a strong impact on post office capacity in the affected municipalities. Note that these clusters could still feature higher *c*_*i*_ values than some found in the here unconsidered rural parts of Wales, since this analysis is only focused on urban areas. In contrast, the municipalities located in the eastern counties of Denbigshire and Flintshire are hotspots and thus offer better temporal access to post office services. Many post offices in the north-eastern urbanised communities are integrated into supermarkets with long opening hours (mainly Spar), often open seven days a week. This is the case for Rhyl and Prestatyn, which are both hotspots in terms of both calculated indicators. Looking south, Fig. 6b shows that there is no comparable directional gradient in the main agglomeration of Wales. Instead, the two largest cities, Swansea and Cardiff, are characterised by a marked difference between their city centres and suburbs. While the city centres appear on the map as coldspots, the suburbs are classified as hotspots. This result explains why these two city centers and the adjacent suburbs appear as attenuated hotspots in the $G_{i}^{*}$ analysis compared to the more consistent other southern population centres. On the other hand, this pattern shows small-scale differences within the cities. Most coldspots in the city centres of Cardiff and Swansea are located in shopping precincts, malls, or even on the campus of Swansea University. Many of these post offices are independent shops, while few are integrated into convenience stores. It is important to note that these city centre offices are likely geared to pedestrian traffic. They have aligned their opening hours with those of nearby high street shops, many of which close at relatively early hours. In contrast, suburban post offices, including those integrated into grocery stores, focus more on car access and therefore often have higher capacity values. The investigation of the temporal capacity measure *c*_*i*_ using two different spatial statistics shows that it is important to consider not only the geospatial accessibility of post offices, but also their (at least potential) service times for basic banking services.

## Conclusions

This paper studies access to basic banking services provided through the post office network in Wales. We reviewed government policies from between 2009 and 2019 that influenced the post office network function as a provider of basic banking services. These policies show that the current model for providing basic banking services is shifting from traditional retail banks to the post office network. The locations and opening hours of post offices were web-scraped from the official website of Post Office Ltd in order to work with an up-to-date data set and to also obtain opening hours. The opening hours were converted into easy-to-interpret temporal capacity scores. These data sets were analysed using two types of methods. One of the methods used was the calculation of geographical access using isochrones, that is, areas that can be reached within certain thresholds and from given coordinates. Both temporal and spatial thresholds were used for this. The calculation of the isochrones was based on the Welsh road network as it is available from OpenStreetMap, and the Openrouteservice routing engine of Heidelberg University was used for the calculations. The second method was to apply two spatial-statistical hotspot measures to give a clearer picture of the temporal capacity indicators used in this study. In addition, we used two different population datasets: a fine-grained population grid provided by the European Union was used for population modelling and the ONS mid-2019 Output Area population. In this way, results based on spatial, temporal and spatio-temporal criteria were achieved. We showed that distance isochrones for the same thresholds as the Post Office access criteria coupled with the capacity of a point of access give us a more refined view of access to the post office network. In these ways, our results obtained provide evidence to inform policy-making in the context of the ongoing transformation of the post office network.

The post office access criteria are often invoked in the retail banking world as a proof that there is national coverage for basic banking services, if not through the banks’ branches then at least through post offices. The coverage of the rural area is particularly important from this point of view as bank branches are usually to be found in areas with higher population density. Our analysis of the post offices by capacity revealed that many of the rural post offices are open only a few hours per week and it is very likely but not certain that these are outreach post offices. The distinction between urban and rural areas deviates from some of the previous findings that were obtained for parts of England (Langford & Higgs, 2010; Ibrahim & Lawal, 2015). Do such potential outreach offices offer the same level of banking services as those with longer opening hours? Can they, for example, accept deposits of thousands of pounds as other post offices can do? These questions remain unanswered in this study because we do not have available the kinds of information necessary to answer them. However, these questions about the equity of access to good and comparable banking service quality should be addressed in future research. We further notice that the list of basic baking services, which are mandatory for the post offices, is quite short. One cannot, for example, make a payment in a post office from their bank account unless it is for a utility company or from a list of accepted businesses. This is another area calling for in-depth follow-up research.

Post Office operates a larger network than all the retail banks put together in the UK (Sonea et al., 2019). While large banks are obliged by the Competition and Market Authority to provide open APIs for their locations and products (Open Banking Implementation Entity, 2017), Post Office seems to be exempt from such a requirement. From our attempt to obtain a data set for the post office locations in Wales it became apparent how changeable the locations and opening hours of the post offices are. Further, the ongoing COVID-19 pandemic led to the temporary closure of many points of access accompanied by restrictions in travelling far away from home. By adopting the UK banking industry standard for making available their locations, opening hours, and services, the Post Office would make it much easier for individuals, local administrators, and policy makers to make decisions on where to go or what critical offices to keep open. These conclusions are supported and underpinned by our results that indicate the problematic pedestrian situation regarding access to Welsh post offices. The current COVID-19 pandemic and the associated temporary closures make visible how important it is to maintain a dense, local, and accessible network of offices offering basic banking services for as many people as possible. Otherwise, the already long distances people have to go will be further exacerbated in future crises.

It is also informative to compare our findings to those obtained by Langford and Higgs (2010). Both our studies confirm that the official access criteria to post offices for Wales are generally not met. However, our method of using both Output Area populations and a 250-m population grid in combination shows a much lower population coverage for all distance thresholds in comparison to the results reported by Langford and Higgs (2010). For example, we demonstrate that only 72.43% of the Welsh population lives within 1 mile reach by car of a post office, substantially less than the 85.30% revealed by Langford and Higgs (2010). What our study cannot afford, however, is a specific distinction between urban and rural using the respective ONS classification. We have revealed urban-rural differences in our spatial analysis but did not take account of the classification in our isochrone analysis. This distinction was taken into account by Langford and Higgs (2010) and has led to interesting insights into variations between those different kinds of statistical units with regards to post office access. Another difference to the Langford and Higgs (2010) study is the way temporal aspects have been considered. Langford and Higgs (2010) have used the official classification of so-called outreach offices while classifying all other offices as full-time. According to our temporal assessments, however, it is questionable whether all those full-time branches are really full-time or can offer basic banking services. It would be interesting for future studies to combine certain aspects of both the previous and our study.

Our study does not take into consideration the ‘demand’ for post office services and more specifically for basic banking services. Very recently Langford et al. (2020) proposed a two-step floating catchment area for calculating supply-to-demand ratios for banking services through banks’ branches in Wales. As banking services are quite diverse in their nature and can be provided through very different points of access (bank branches, post offices, ATMs, cashpoints) we suggest to consider all these points of access together but separate the demand by type of service (that is, cash, business banking). As bank branch, Post Office, and ATM networks are under different pressure forces to reduce their numbers, while the national coverage remains a necessity of public interest, spatial optimisation methods (e.g., Cao et al., 2020) should be explored in more detail and in regard to the topic discussed in this article for determining possible optimal coverage structures of these overlapping points of access to basic banking services. As stated, this study is limited to the supply side of post office-based provision of banking services, which is of great importance for the settlement of companies, future residents, and so forth. Given that demand modelling is very important too, but a complex task that goes beyond the scope of this paper, we strongly recommend that it is taken into account in future studies, in order to obtain further and timely insights on the issues discussed in this article from a different, complementary and important perspective. Greater attention to the demand side will be particularly helpful in further exploring the role and utility of mobile branches, which our research has identified as a potential driving force for the emergence of statistical coldspots with regards to temporal capacity.

## Footnotes

1 https://www.postoffice.co.uk/all-locations; the website has changed since we scraped our data set

2 https://www.ons.gov.uk/methodology/geography/ukgeographies/censusgeography; last accessed 8 December 2020

3 https://www.ons.gov.uk/peoplepopulationandcommunity/populationandmigration/populationestimates/datasets/censusoutputareaestimatesinwales; last accessed 8 December 2020
